# The neural crest transcription factor Brn3a is expressed in melanoma and required for cell cycle progression and survival

**DOI:** 10.1002/emmm.201201862

**Published:** 2013-05-13

**Authors:** Tobias Hohenauer, Carola Berking, Andreas Schmidt, Sebastian Haferkamp, Daniela Senft, Claudia Kammerbauer, Sabine Fraschka, Saskia Anna Graf, Martin Irmler, Johannes Beckers, Michael Flaig, Achim Aigner, Sabrina Höbel, Franziska Hoffmann, Heiko Hermeking, Simon Rothenfusser, Stefan Endres, Thomas Ruzicka, Robert Besch

**Affiliations:** 1 Department of Dermatology and Allergology, Ludwig-Maximilian UniversityMunich, Germany; 2 Division of Clinical Pharmacology, Department of Internal Medicine, Ludwig-Maximilian UniversityMunich, Germany; 3 Center of Integrated Protein Science CIPS-M at the Division of Clinical Pharmacology, Department of Internal Medicine, Ludwig-Maximilian UniversityMunich, Germany; 4 Department of Dermatology, Venereology and Allergology, University Hospital WürzburgWürzburg, Germany; 5 Helmholtz Zentrum München, German Research Center for Environmental Health, Institute of Experimental GeneticsNeuherberg, Germany; 6 Technical University Munich, Center of Life and Food Sciences WeihenstephanFreising, Germany; 7 Institute of Pharmacology, Philipps-University, Faculty of MedicineMarburg, Germany; 8 Experimental and Molecular Pathology, Institute of Pathology, Ludwig-Maximilian UniversityMunich, Germany; 9 Section of Gastroenterology and Endocrinology, Medizinische Klinik Innenstadt, Ludwig-Maximilian UniversityMunich, Germany

**Keywords:** apoptosis, DNA damage, melanoma, neural crest factors, tumourigenesis

## Abstract

Pigment cells and neuronal cells both are derived from the neural crest. Here, we describe the Pit-Oct-Unc (POU) domain transcription factor Brn3a, normally involved in neuronal development, to be frequently expressed in melanoma, but not in melanocytes and nevi. RNAi-mediated silencing of Brn3a strongly reduced the viability of melanoma cell lines and decreased tumour growth *in vivo*. In melanoma cell lines, inhibition of Brn3a caused DNA double-strand breaks as evidenced by Mre11/Rad50-containing nuclear foci. Activated DNA damage signalling caused stabilization of the tumour suppressor p53, which resulted in cell cycle arrest and apoptosis. When Brn3a was ectopically expressed in primary melanocytes and fibroblasts, anchorage-independent growth was increased. In tumourigenic melanocytes and fibroblasts, Brn3a accelerated tumour growth *in vivo*. Furthermore, Brn3a cooperated with proliferation pathways such as oncogenic BRAF, by reducing oncogene-induced senescence in non-malignant melanocytes. Together, these results identify Brn3a as a new factor in melanoma that is essential for melanoma cell survival and that promotes melanocytic transformation and tumourigenesis.

## INTRODUCTION

Characteristic properties of cancer cells, such as increased cell proliferation, survival or cell migration, are also essential for embryonic development. Re-expression of molecules involved in embryogenesis can confer malignant properties to tumour cells. Lineage-specific re-expression of such factors can characterize certain cancer types and is of potential therapeutic value.

Melanoma, one of the deadliest types of skin cancer, derives from melanocytes. Melanocytes and neuronal cells both originate from the same precursor cells located in the neural crest. During embryonic development, multipotent neural crest cells become progressively restricted to specific sub-lineages, including neuronal cell types, endocrine cells and melanocytic cells (Crane & Trainor, 2006). In neuroectodermal tumours as well as in melanoma, a deregulated expression of neuronal factors normally expressed in neural crest cells may contribute to malignancy (Herlyn et al, 2000; Pla et al, 2001; Truzzi et al, 2008).

The transcription factors of the Pit-Oct-Unc (POU) domain family play important roles in the development of neuronal cells. Brn2 (*POU3F2*) is required for the development of endocrine tissues and neurosecretory neurons (Schonemann et al, 1995), and tumour-promoting effects have been described (Carreira et al, 2005; Cook & Sturm, 2008; Goodall et al, 2008). Brn3a (*POU4F1*) is expressed in proliferating precursor cells of the peripheral nervous system originating from the neural crest (Fedtsova & Turner, 1995). In adults, Brn3a expression is primarily restricted to neuronal tissues, *i.e.* sensory ganglia and parts of the central nervous system (Collum et al, 1992; He et al, 1989). More recently, Brn3a expression was detected also in other tissues (Budhram-Mahadeo et al, 2001, 1999b). Brn3a-deficient mice display apoptosis of certain sensory neurons (McEvilly et al, 1996; Xiang et al, 1996). Tumour-promoting potential of Brn3a has been proposed, because of this antiapoptotic role in neuronal cells and a structural similarity to c-myc (Ensor et al, 2001; Gerrero et al, 1993). Brn3a can activate transcription of antiapoptotic Bcl-2 and Bcl-xL by binding to their respective promoters (Ensor et al, 2001; Smith et al, 1998, 2001). Furthermore, Brn3a can interact with the tumour suppressor p53 and this protein–protein interaction seems to modulate the activity of both factors (Budhram-Mahadeo et al, 1999a; Hudson et al, 2005).

Here, we demonstrate expression of Brn3a in melanoma. Brn3a is of high relevance for melanoma cell proliferation and survival. Furthermore, in non-malignant cells expression of Brn3a promotes malignant transformation.

## RESULTS

### Brn3a is expressed in human melanoma, but not in melanocytes and other non-malignant skin cells

**Figure 1:**
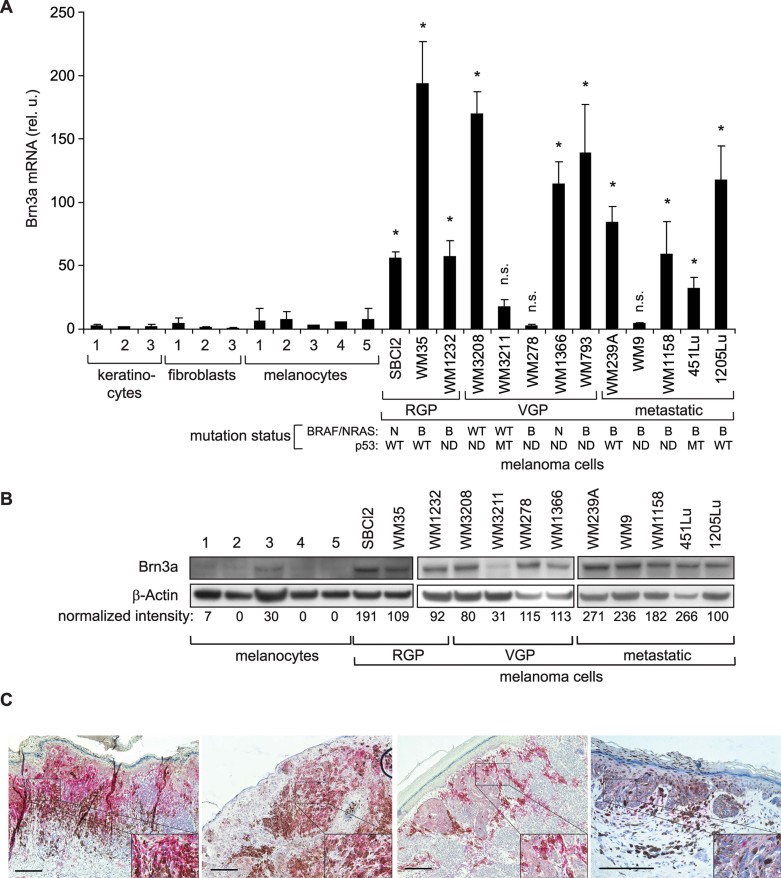
Brn3a is expressed in human melanoma, but not in melanocytes and other non-malignant skin cells Brn3a mRNA levels in primary human skin cells of different donors and human melanoma cell lines (RGP, radial growth phase; VGP, vertical growth phase). Mean ± SD is shown. The mutation status of BRAF/NRAS and of p53 is indicated (N: NRAS mutated, B: BRAF mutated, WT: wild-type, MT: p53 mutated, ND: mutation status not determined); *p < 0.05 *versus* all melanocyte donors, *t*-test, *n* = 3 per group; n.s.: not significant (*p* > 0.05).Brn3a protein levels were determined by immunoblotting using an antibody that detects full-length Brn3a isoform (47 kDa). 1205Lu cells were included on each blot to ensure equal film exposure (not shown). Representative blots (*n* = 3) are shown.Specimens of human primary melanomas were analysed for Brn3a expression *in situ* by immunohistochemistry. Four representative samples are shown. Red staining represents Brn3a, brownish colour pigment. Scale bar: 200 µm. The lower-right insert depicts a fourfold magnification of the respective area.

Brn3a expression was analysed in human melanoma cell lines and in non-malignant skin cells. More than 75% of melanoma cell lines (10 of 13) expressed highly increased levels of Brn3a mRNA compared to melanocytes, fibroblasts and keratinocytes (Fig 1A). At the protein level, Brn3a was increased in almost all melanoma cell lines (11 of 12) and was not associated with a specific progression stage (Fig 1B). In WM9 and WM278 cells, Brn3a protein was clearly detectable despite low mRNA levels. The regulation of Brn3a in these cells is not entirely clear. Only one cell line (WM3211) was found to express low amounts of Brn3a both on mRNA and on protein level. In human tissue, Brn3a was detected in 55% (49 of 89) primary melanoma samples (Fig 1C). The intensity of staining ranged from strong to rather weak, and both large tumour areas with homogenous staining as well as small Brn3a-positive areas were observed. Strong activity of a Brn3a luciferase reporter was observed in Brn3a-expressing melanoma cell lines (1205Lu, WM1158, WM1232), but not in WM3211 cells with low Brn3a levels (Supporting Information Fig S1A) confirming transcriptional activity of Brn3a in melanoma. Successful transfection of siRNA and expression vectors was confirmed in this cell line, which therefore was used as negative control in subsequent experiments (Supporting Information Fig S1B).

### Inhibition of Brn3a reduces melanoma cell viability and leads to reduced tumour growth *in vivo*

**Figure 2:**
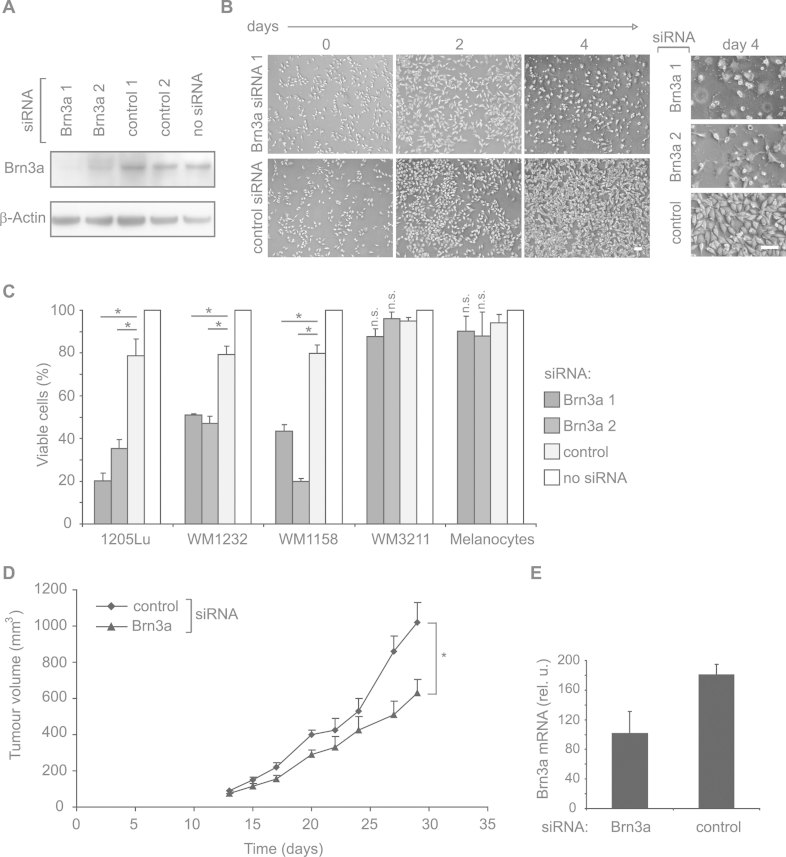
Inhibition of Brn3a reduces melanoma cell viability and leads to reduced tumour growth *in vivo* 1205Lu melanoma cells were transfected with two siRNAs specific for Brn3a (Brn3a 1 and Brn3a 2) or control siRNAs and analysed by immunoblotting 48 h after transfection. Representative blots (*n* = 3) are shown.Microscopic pictures of 1205Lu melanoma cells 2 or 4 days after transfection of Brn3a-specific siRNAs or control siRNA. Scale bars: 100 µm.Different melanoma cell lines, *i.e.* 1205Lu, WM1158 and WM1232 (high Brn3a levels) and WM3211 (low Brn3a levels), were transfected with siRNAs as described in (A). Viable melanoma cells were quantified 4 days after siRNA transfection. Viability of cells treated with transfection reagent alone (‘no siRNA’) was set to 100%. Mean ± SD is shown. *p = 0.003 or less, *t*-test, *n* = 3 per group; n.s.: not significant (*p* > 0.05).1205Lu melanoma cells were subcutaneously injected into nude mice. Upon palpability of tumours, mice were systemically treated three times per week by intraperitoneal injection of 10 µg of Brn3a-specific or control siRNA complexed with polyethylenimine. Tumour growth in Brn3a siRNA-treated animals was significantly reduced (*p* = 0.0078, Wilcoxon matched pair test, *n* = 5 per group).Brn3a mRNA levels in tumours isolated at day 29. Mean of each group ± SEM is shown.

The functional relevance of Brn3a in melanoma was studied using RNAi. Two siRNAs targeting Brn3a were used in order to control for non-specific effects. Both efficiently inhibited Brn3a expression (Fig 2A) and transcriptional activity (Supporting Information Fig S1C). Silencing of Brn3a resulted in a reduced cell number and altered morphology (Fig 2B). Reduced cell viability (more than 50%) was confirmed in several Brn3a-expressing melanoma cell lines, but not in WM3211 cells (low Brn3a levels) or primary melanocytes (Fig 2C). In general, siRNA transfection into melanocytes was successful but less reliable compared to melanoma cells. Therefore, it cannot be excluded that low inhibition efficacy is one reason for unaltered melanocyte viability. Next, we tested whether Brn3a inhibition affects tumour growth *in vivo*. Nude mice carrying palpable subcutaneous tumours were systemically treated with siRNA. Brn3a inhibition significantly delayed tumour growth (Fig 2D). Brn3a mRNA in tumours was reduced by around 50%, indicating successful targeting of Brn3a (Fig 2E).

### Brn3a inhibition induces cell cycle arrest followed by apoptosis in melanoma cells

**Figure 3:**
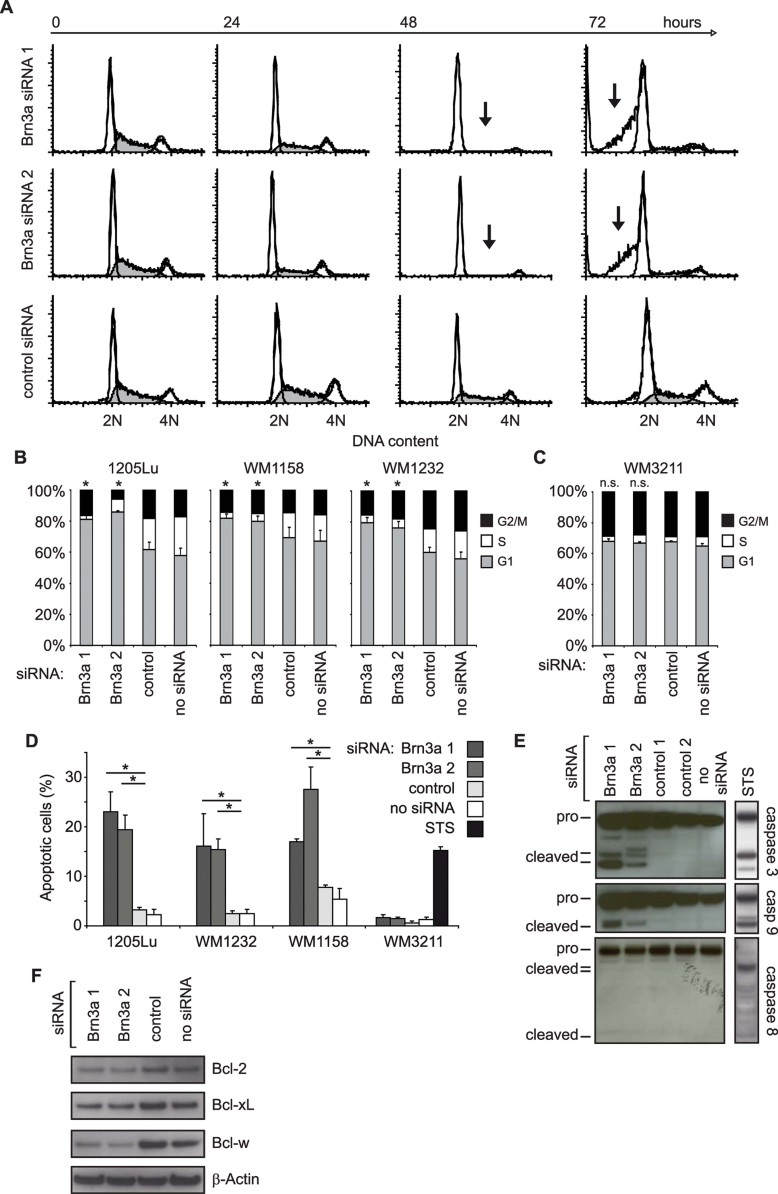
Inhibition of Brn3a leads to cell cycle arrest in melanoma cells followed by mitochondrial apoptosis Cell cycle analysis of 1205Lu melanoma cells 24, 48 and 72 h after transfection with Brn3a-specific or control siRNAs. Arrows indicate the loss of cells in S and G2 phase at 48 h and the sub-G1 fraction typical for apoptosis at 72 h. Representative histograms (*n* = 3) are shown.Cell cycle analysis of 1205Lu, WM1158 and WM1232 melanoma cells treated as described in (A). The mean of cells in G1 (grey), S (white) and G2/M phase (black) is shown. Error bars represent SD of cells in G1 phase. *p = 0.029 or less compared to control siRNA-treated cells, *t*-test, *n* = 3 (1205Lu), 4 (WM1158) and 5 (WM1232); n.s.: not significant (*p* > 0.05).Cell cycle analysis of the cell line WM3211 (low Brn3a levels) as described in (B).Analysis of apoptosis in 1205Lu, WM1158, WM1232 and WM3211 melanoma cells 4 days after transfection of Brn3a-specific or control siRNAs. Annexin V-positive and propidium iodide-negative cells are depicted. Mean ± SD is shown. *p = 0.022 or less, *t*-test, *n* = 3 per group. Treatment with staurosporine (STS) served as positive control for apoptosis induction in WM3211.Analysis of caspase 3, 9 and 8 activation in 1205Lu cells treated as described in (D) by immunoblotting with antibodies specific for procaspases (pro) and their respective active subunits (cleaved). WM9 melanoma cells treated with 1 µM staurosporine (STS) for 5 h served as a positive control. Representative blots (*n* = 3) are shown.Quantification of antiapoptotic Bcl-2, Bcl-xL and Bcl-w by immunoblotting 3 days after transfection of Brn3a-specific or control siRNAs. Representative blots (*n* = 3) are shown.

To explore the cause of reduced cell viability, cell cycle analysis was performed. A striking loss of 1205Lu cells in the S phase became apparent 48 h after Brn3a inhibition (Fig 3A). Similarly, a loss of cells in the S phase and an increase in the G1 phase was detected in WM1158 and WM1232 cells (Fig 3B), but not in WM3211 cells (Fig 3C). To further control that cell cycle arrest is indeed due to loss of Brn3a, we generated mutant Brn3a variants that contain silent base pair mismatches within the siRNA binding site (Supporting Information Fig S1D). Whereas Brn3a inhibition was still present upon overexpression of wild-type Brn3a, ectopic expression of the respective mutant form compensated for endogenous Brn3a losses induced by the corresponding siRNA (Supporting Information Fig S1E). Cell cycle analysis revealed that melanoma cells were rescued from Brn3a siRNA-mediated cell cycle arrest when the corresponding mutant Brn3a variant was expressed (Supporting Information Fig S1F). This indicates that the cell cycle arrest was indeed due to loss of Brn3a.

Later (72 h), a sub-G1 fraction suggested that these cells undergo apoptosis (Fig 3A). This was confirmed by Annexin V staining showing an increase in apoptotic cells to around 20% (Fig 3D). Again, apoptosis was not induced in WM3211 cells (Fig 3D) and in melanocytes. Caspase 3 and 9, but not caspase 8, were cleaved indicating activation of the mitochondrial apoptosis pathway (Fig 3E). Likewise, release of cytochrome *c* into the cytosol was observed (Supporting Information Fig S2A). In addition, antiapoptotic Bcl-2, Bcl-xL and Bcl-w levels were reduced (Fig 3F) and proapoptotic Bax and Bak levels enhanced (Supporting Information Fig S2B). On transcript level, Bcl-2 and Bcl-xL were reported to be transcriptionally upregulated by Brn3a (Smith et al, 1998, 2001), however, in melanoma, Bcl-xL mRNA was not altered upon Brn3a inhibition and Bcl-2 mRNA levels were indirectly regulated by activated p53 (see below) as evidenced by co-inhibition studies (Supporting Information Fig S2C). Similarly, proapoptotic molecules Bax and Bak were found to be regulated via p53 (Supporting Information Fig S2D).

### The tumour suppressor p53 is activated upon Brn3a inhibition and mediates cell cycle arrest

**Figure 4:**
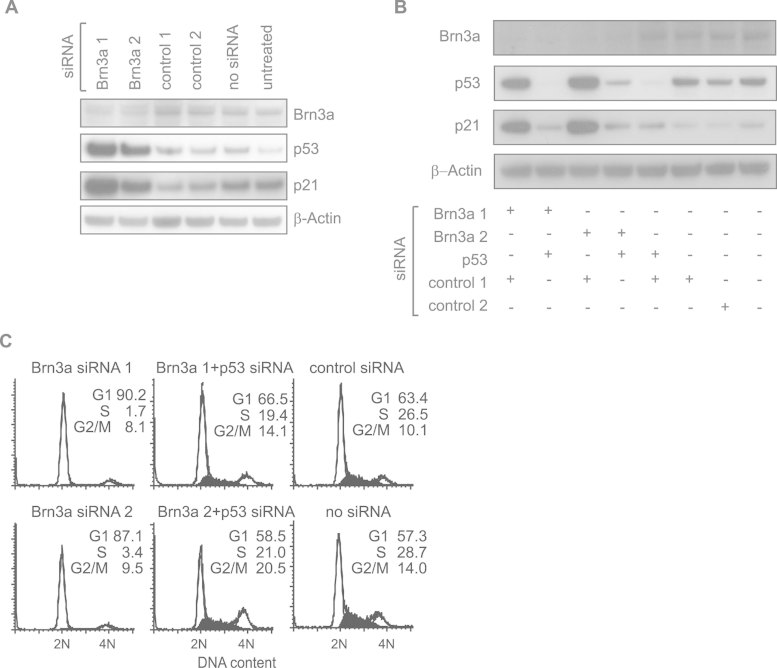
The tumour suppressor p53 is activated upon Brn3a inhibition and mediates cell cycle arrest Analysis of Brn3a, p53 and p21 by immunoblotting in 1205Lu melanoma cells 48 h after transfection of Brn3a-specific or control siRNAs. Representative blots (*n* = 3) are shown.Analysis of Brn3a, p53 and p21 in 1205Lu cells 48 h after co-transfection of Brn3a-specific, p53-specific or control siRNAs. Representative blots (*n* = 3) are shown.Cell cycle analysis of 1205Lu cells treated as described in (B). Representative histograms (*n* = 3) are shown. The percentage of cells in the G1, S and G2/M phase is indicated in the upper right part of the histogram.

Immunoblots revealed a strong increase in p53 levels upon Brn3a inhibition, associated with increased p21, a cell cycle inhibitory protein and known p53 target gene in melanoma (Besch et al, 2007) (Fig 4A). Increased p53 levels were associated with increased activity as determined by DNA binding activity (Supporting Information Fig S3A). p53 accumulated due to decreased protein turnover (Supporting Information Fig S3B). Next, the relevance of p53 was tested by blocking p53 activation in Brn3a-inhibited cells. Co-transfection of 1205Lu cells with p53- and Brn3a-specific siRNA efficiently abrogated accumulation of p53 and consequently blocked induction of p21 (Fig 4B). Cell cycle analysis revealed that cell cycle progression was clearly restored when p53 was blocked, indicating that p53 was indeed responsible for the cell cycle arrest upon Brn3a inhibition (Fig 4C and Supporting Information Fig S3C).

### Loss of Brn3a in melanoma leads to DNA double-strand breaks and activation of DNA damage signalling

**Figure 5:**
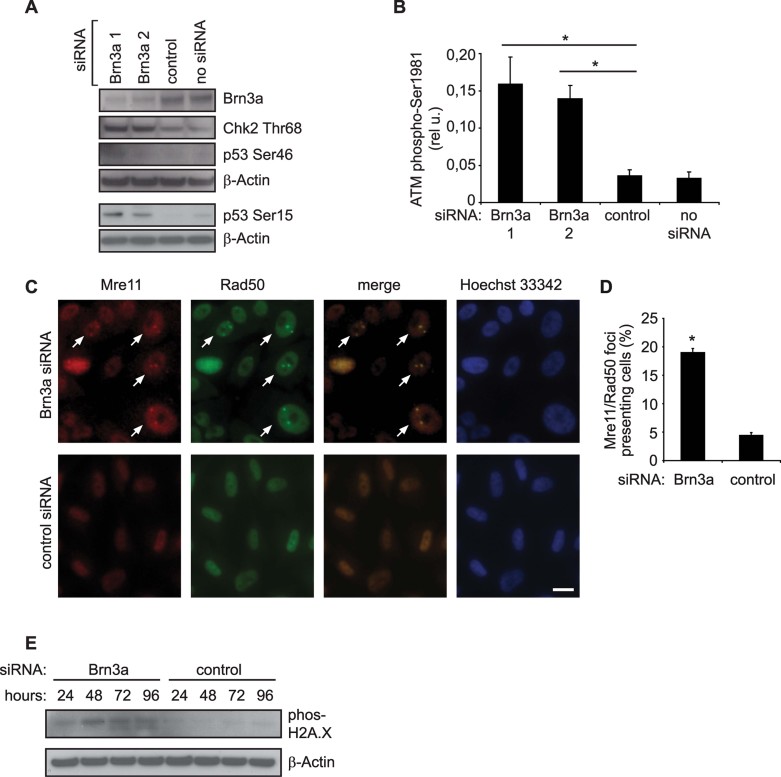
Inhibition of Brn3a causes DNA double-strand breaks that lead to activation of p53 Analysis of p53 phosphorylation (Ser15 and Ser46) and of checkpoint kinase 2 (Chk2; Thr68) in 1205Lu melanoma cells by immunoblotting 48 h after transfection of Brn3a-specific or control siRNA. Representative blots (*n* = 3) are shown.Phosphorylation status of ATM (Ser1981) by ELISA in 1205Lu cells treated as described in (A). Mean ± SD is shown. *p < 0.0009, *t*-test, *n* = 6 per group.Analysis of Mre11/Rad50-containing enzyme foci, representing active sensing of DNA double-strand breaks, in 1205Lu cells 40 h after transfection of Brn3a-specific or control siRNA. Immunofluorescence staining of Mre11 (red), Rad50 (green) and of nuclei (Hoechst 33342; blue) is shown. Arrows indicate nuclei with Mre11/Rad50 foci. Representative results (*n* = 3) are shown. Scale bar: 50 µm.Percentage of cells containing Mre11/Rad50 foci treated as described in (C). Mean ± SD is shown. *p < 0.0009, *t*-test, *n* = 3 per group.Time-course analysis of histone H2A.X phosphorylation. Immunoblots were performed 24, 48, 72 and 96 h after Brn3a siRNA transfection. Representative blots (*n* = 2) are shown.

It has been suggested that Brn3a and p53 can interact on the protein level, thereby antagonizing transcriptional activity of both molecules (Budhram-Mahadeo et al, 1999a; Hudson et al, 2005; Sugars et al, 2001). Hence Brn3a could be an important suppressor of p53 in melanoma. However, in melanoma cells, we could not detect interaction of Brn3a protein with endogenous p53 in co-immunoprecipitation assays. In addition, increased p53 protein levels are not expected when p53 is activated due to release from Brn3a interaction. To explore alternative mechanisms, we analysed p53 phosphorylation patterns. p53 phosphorylation at Ser15, but not at Ser46, was observed upon silencing of Brn3a (Fig 5A), consistent with activated DNA damage signalling (Shieh et al, 1997). This was confirmed by increased phosphorylation of Chk2 (Fig 5A) and of ataxia teleangiectasia mutated (ATM) (Fig 5B). Next, nuclear Mre11/Rad50-containing foci were analysed, which are formed on DNA double-strand breaks (Hopfner et al, 2002). A significant increase (from 5% in controls to 20%) in foci-containing cells was observed upon Brn3a inhibition (Fig 5C,D). DNA damage was also confirmed by staining cells for histone H2A.X phosphorylation in a time course experiment, where H2A.X phosphorylation became apparent after 48 h (Fig 5E), coinciding with the onset of cell cycle arrest. Together, the data demonstrate that loss of Brn3a in melanoma causes DNA damage that leads to p53 activation.

### Brn3a promotes anchorage-independent growth *in vitro* and tumour growth *in vivo*

**Figure 6:**
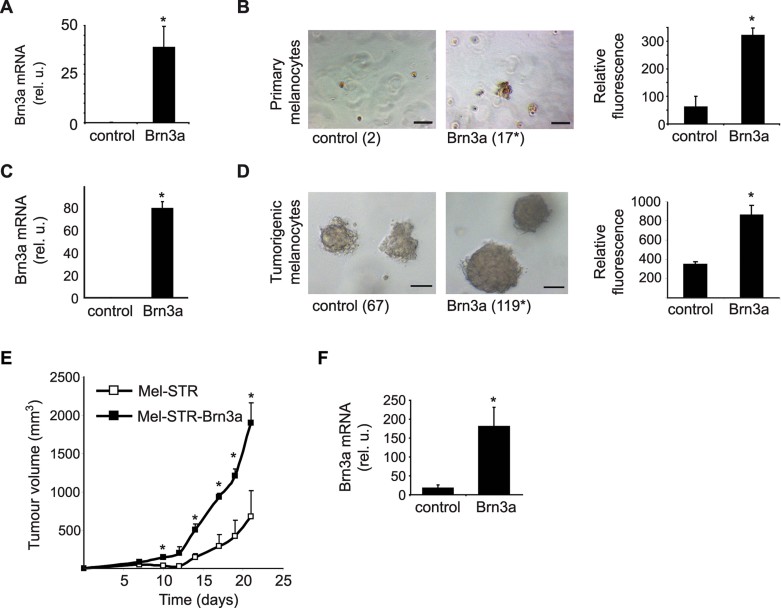
Brn3a promotes anchorage-independent growth *in vitro* and tumour growth *in vivo* Brn3a levels in primary human melanocytes after lentiviral transduction of a Brn3a-encoding or an empty vector (control). Mean ± SD is shown. *p = 0.023, *t*-test, *n* = 3 per group.Left panel: Microscopic quantification of colony formation of primary melanocytes cultured for 11 days in soft agar. Numbers of colonies (>10 cells) are shown in brackets. *p = 0.001, *t*-test, *n* = 3 per group. Right panel: Fluorimetric quantification of cellular DNA of cells cultured for 9 days in soft agar. Mean ± SD is shown. *p = 0.001, *t*-test, *n* = 3 per group.Brn3a levels in tumourigenic Mel-STR melanocytes that were lentivirally transduced with Brn3a-encoding (Brn3a) or empty (control) vectors. Mel-STR melanocytes are tumourigenic due to defined genetic alterations. Mean ± SD is shown. *p = 0.003, *t*-test, *n* = 2 per group.Left panel: Colony formation of Mel-STR melanocytes at day 7. *p = 0.007, *t*-test, *n* = 3 per group. Right panel: Fluorimetric quantification of anchorage-independent growth. Mean ± SD is shown. *p = 0.009, *t*-test, *n* = 3 per group.Subcutaneous tumour growth of Brn3a-expressing or control Mel-STR melanocytes. Mean ± SEM is shown. *p < 0.05, *t*-test, *n* = 3 per group.Brn3a mRNA levels in tumours at the end of the experiment. Mean ± SD is shown. *p = 0.006, *t*-test, *n* = 3 per group.

Altogether, the data demonstrated an essential role of Brn3a in melanoma cells. We, next, tested the effects of Brn3a in skin cells, which display low Brn3a expression levels (Fig 1A and B). First, Brn3a was expressed in primary human melanocytes by lentiviral transduction (Fig 6A). Potential oncogenic properties of Brn3a were assessed by determining anchorage-independent growth. Colony formation was significantly enhanced in melanocytes expressing Brn3a, resulting in a fivefold increased cell growth in soft agar (Fig 6B). Similar results were achieved with immortalized melanocytes (Supporting Information Fig S4). Notably, similar observations were made in primary human fibroblasts, which confirms the oncogenic role of Brn3a irrespective of the cell lineage (Supporting Information Fig S5A and B). When subcutaneously inoculated into NOD-SCID mice, these cells did not form tumours indicating that further factors besides Brn3a are required to convert primary cells into tumourigenic cells. To further test the role of Brn3a *in vivo*, Brn3a was analysed in immortalized NIH3T3 fibroblasts, known to form tumours solely upon expression of HRAS (Land et al, 1983). HRAS-transformed NIH3T3 fibroblasts formed colonies in soft agar. Co-expression with Brn3a, however, almost doubled colony formation and cell growth (Supporting Information Fig S5C and D). *In vivo*, tumour growth of HRAS-transformed NIH3T3 was considerably increased upon Brn3a expression (Supporting Information Fig S5E). Increased levels of Brn3a in tumours were confirmed after animals were sacrificed (Supporting Information Fig S5F). Finally, as a model more closely related to melanoma, primary human melanocytes were analysed that had been engineered to become tumourigenic by defined genetic modifications, *i.e.* hTERT, large T antigen and HRAS (Gupta et al, 2005). Again, Brn3a expression doubled colony numbers and cell growth in soft agar assays, and promoted tumour growth *in vivo* (Fig 6C–F). Together, the data provide evidence for an oncogenic role of Brn3a, as Brn3a promotes anchorage-independent growth *in vitro* and tumour growth *in vivo*.

### Brn3a cooperates with activated RAS/RAF signalling by reducing oncogene-induced senescence in melanocytic tumourigenesis

**Figure 7:**
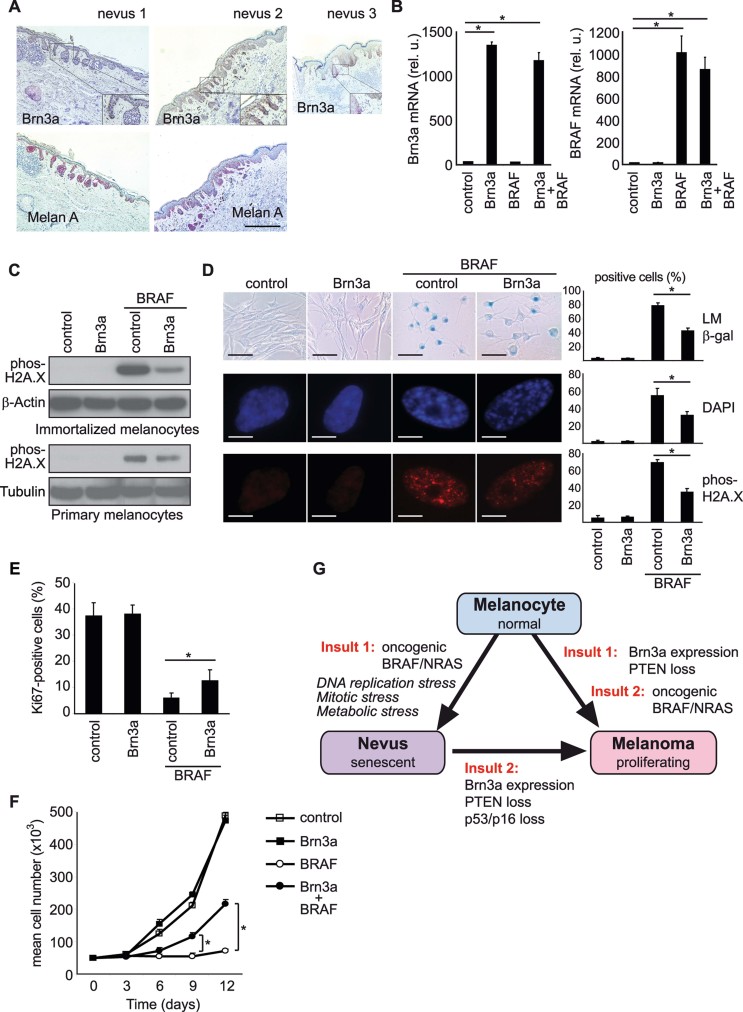
Brn3a reduces oncogene-induced senescence in melanocytes Thirty-eight specimens of human melanocytic nevi were analysed for Brn3a expression *in situ* by immunohistochemistry. Two representative samples stained for Brn3a (upper panel) or the melanocyte marker Melan A (lower panel) are shown. Nevus 3 (right panel) represents the Brn3a-positive nevus found in this cohort. Scale bar: 200 µm. The lower-right insert depicts a fourfold magnification of the respective area.Expression of Brn3a (left panel) and ^V600E^BRAF (right panel) in immortalized (p16 null, hTERT overexpressing) human melanocytes that were transduced with an empty vector (control), Brn3a-encoding vector (Brn3a), or co-transduced with ^V600E^BRAF- and Brn3a-encoding vectors (Brn3a + BRAF). Mean ± SD is shown. *p = 0.012 or less, *t*-test, *n* = 2 per group.Analysis of DNA damage by immunoblotting of phosphorylated histone H2A.X in Brn3a-, ^V600E^BRAF- or Brn3a/^V600E^BRAF-expressing immortalized (upper panel) or primary human melanocytes (lower panel). Representative blots (*n* = 3) are shown.Primary human melanocytes were transduced with empty vector (control), ^V600E^BRAF alone (BRAF), or ^V600E^BRAF together with Brn3a. Cells were analysed at day 6 for the appearance of markers indicative for senescence: β-galactosidase activity (β-gal) was visualized by light microscopy (LM). Scale bar: 100 µm. Heterochromatin foci (DAPI) and phosphorylated H2A.X foci (phos-H2A.X) were assessed by immunofluorescence microscopy. Representative cell nuclei are shown. Scale bar: 10 µm. Right panel: Mean percentage of cells displaying increased β-galactosidase activity (*p < 0.0009, *t*-test, *n* = 4 per group), containing heterochromatin foci (*p < 0.003, *t*-test, *n* = 4 per group), or containing phos-H2A.X foci (*p < 0.014, *t*-test, *n* = 2 per group). Mean ± SD is shown.Percentage of Ki67-positive melanocytes that were treated as described in (D). Mean ± SD is shown. *p = 0.026, *t*-test, *n* = 4 per group.Primary melanocytes transduced as described in (D). Two days after transduction, 50,000 cells were seeded (day 0). Cell number was determined by counting at day 3, 6, 9 and 12 after seeding. Mean ± SD is shown. *p = 0.002 or less, *t*-test, *n* = 3 per group.Summary of the potential role of Brn3a in tumourigenesis of melanoma with respect to known oncogenic insults.

Brn3a was associated with malignant transformation and may cooperate with active proliferation drivers common in tumours. Indeed, Brn3a promoted tumour growth in synthetic tumour models where RAS signalling is activated (Fig 6C–F, Supporting Information Fig S5C-F). In primary cells, however, proliferation drivers such as oncogenic RAS or RAF trigger a permanent cell cycle arrest termed oncogene-induced senescence, which is considered to be a cellular defence mechanism that protects against transformation. Brn3a was detected even in early stage melanoma cell lines, but not in melanocytes (Fig 1A and B). In nevi – benign melanocytic tumours – the activating ^V600E^BRAF point mutation is found in up to 80%. It has been suggested that a senescence growth arrest is induced in these common melanocytic lesions thereby preventing progression to malignant melanoma (Michaloglou et al, 2005; Pollock et al, 2003). Furthermore, there is compelling evidence that expression of ^V600E^BRAF in primary human melanocytes causes senescence *in vitro* (Gray-Schopfer et al, 2006; Michaloglou et al, 2005) indicating that mutated BRAF alone is not sufficient for malignant transformation to melanoma. To investigate whether Brn3a plays a role in the regulation of ^V600E^BRAF-induced melanocyte senescence and melanomagenesis, we analysed benign melanocytic nevi for Brn3a expression. Notably, only 3% (1 of 38) stained positive, compared to 55% (49 of 89) in primary melanoma samples, indicating that Brn3a is a clear indicator of benign *versus* malignant lesions (Fig 7A). The low abundance of Brn3a in nevi led to the suggestion that Brn3a might be involved in bypassing the ^V600E^BRAF-induced melanocyte senescence response. DNA double-strand breaks have been implicated in oncogene-induced senescence (Bartkova et al, 2006; Di Micco et al, 2006). Consequently, the DNA double-strand breaks in Brn3a-inhibited melanoma cells could potentially reflect oncogene-induced DNA damage that is not suppressed by Brn3a anymore. Therefore, we expressed Brn3a alone or together with ^V600E^BRAF in melanocytes (Fig 7B). As reported in previous studies, ^V600E^BRAF expression led to DNA damage as determined by histone H2A.X phosphorylation. Importantly, DNA damage was clearly decreased in the presence of Brn3a in immortalized (Sviderskaya et al, 2003) or primary human melanocytes (Fig 7C). Next, the influence of Brn3a on ^V600E^BRAF-driven senescence was studied in more detail. Primary human melanocytes were engineered to express hTERT via lentiviral transduction prior to further investigation to avoid telomere-associated replicative senescence. On day 6 after transduction with ^V600E^BRAF, the oncogene-induced senescence phenotype became fully apparent. Increased numbers of melanocytes displaying increased senescence-associated β-galactosidase activity, senescence-associated heterochromatin foci and foci positive for phosphorylated histone H2A.X were observed (Fig 7D). Consistent with our hypothesis, ^V600E^BRAF-induced senescence was clearly attenuated when Brn3a was expressed along with ^V600E^BRAF. The number of β-galactosidase-positive cells was significantly reduced from 79 to 43%. Similar observations were made when measuring heterochromatin foci and DNA damage-associated foci (Fig 7D). To test whether reduction of senescence markers is indeed associated with increased cell proliferation, cells were stained for the proliferation marker Ki67. ^V600E^BRAF transduction strongly reduced Ki67-positive cells from 37 to 6%. Co-transduction of ^V600E^BRAF and Brn3a, however, led to a significant increase in Ki67-positive cells from 6 to 13% (Fig 7E). When cell proliferation was measured in a time-course experiment, ^V600E^BRAF transduction led to a robust cell proliferation stop that was maintained for at least 12 days. Importantly, Brn3a transduction partially reverted the ^V600E^BRAF-mediated cell proliferation stop. The cells started to proliferate, leading to 3-fold increased cell numbers in ^V600E^BRAF/Brn3a-transduced cells compared to cells transduced with ^V600E^BRAF (Fig 7F). Together, the data provide evidence that Brn3a is absent in senescent nevi, but present in melanoma. Brn3a cooperates with active RAS/RAF signalling by attenuating oncogene-induced DNA damage. Therefore, Brn3a promotes tumourigenesis by reducing oncogene-induced senescence.

## DISCUSSION

Melanocytes and neuronal cells originate from the same precursors in the neural crest. Here, we demonstrate that the POU domain transcription factor Brn3a, normally expressed in neural crest cells, is expressed in melanoma. Compared to other alterations in melanoma, increased Brn3a levels were observed in melanoma at a high incidence (55%) and in the majority of melanoma cell lines, but not in melanocytes and nevi. Brn3a is essential for melanoma cell proliferation and survival: specifically in Brn3a-positive melanoma cells, inhibition of Brn3a causes DNA double-strand breaks leading to p53-mediated cell cycle arrest and subsequent apoptosis. *In vivo*, systemic targeting of Brn3a reduced growth of subcutaneous melanomas.

Furthermore, gain-of-function experiments in Brn3a-negative cells revealed an oncogenic role of Brn3a. Two experimental settings were utilized: In the first setting, Brn3a promoted anchorage-independent growth of non-malignant cells and accelerated tumour growth of tumourigenic cells, demonstrating a general tumour-promoting role of Brn3a. In the second setting, Brn3a was studied in combination with ^V600E^BRAF, the most important oncogenic alteration in melanoma, which is present also in nevi. Here, Brn3a reduced ^V600E^BRAF-induced senescence. This demonstrates that Brn3a, absent in senescent nevi but present in melanoma, may be an important endogenous suppressor of the ^V600E^BRAF-induced melanocyte senescence response and implicates a role of Brn3a in melanocytic tumourigenesis.

In the neural crest, Brn3a is expressed in precursors that differentiate into sensory neurons (Greenwood et al, 1999). Brn3a levels were increased in differentiating cells of the CNS, but Brn3a is also expressed in undifferentiated cell populations (Hudson et al, 2004) and in proliferating cells of the PNS that display mitotic figures (Fedtsova & Turner, 1995). In the melanocytic lineage, a potential role of Brn3a in development has not been determined. Similar to neuronal cells, in which Brn3a expression is terminated in most adult tissues, Brn3a expression was not observed in melanocytes. There is clear evidence that Brn3a is important for neuronal survival. Brn3a-deficient mice experience a massive loss of sensory neurons during embryogenesis (McEvilly et al, 1996; Xiang et al, 1996). Nevertheless, re-expression of Brn3a in tumours has been reported rarely, which may be due to its tissue-specific expression (Collum et al, 1992; Diss et al, 2006; Leblond-Francillard et al, 1997; Ndisdang et al, 1998). In neuronal cells, Brn3a can activate transcription of antiapoptotic genes like Bcl-2 or Bcl-xL (Smith et al, 1998, 2001) and enhance apoptosis resistance (Hudson et al, 2004; Smith et al, 2001). In melanoma cells, besides cell cycle arrest, loss of Brn3a was associated with apoptosis and with decreased Bcl-2 and Bcl-xL levels. However, downregulation of these proteins in response to Brn3a was not observed on the mRNA level, suggesting that both were not directly regulated by Brn3a in melanoma.

A well-documented function of Brn3a is antagonizing p53, which prompted us to study whether Brn3a is a lineage-specific suppressor of p53 in melanoma. In a neuronal cell line, the p53-antagonizing effect of Brn3a was reported to be mediated through protein–protein interactions at the promoter of the respective target gene (Budhram-Mahadeo et al, 1999a; Hudson et al, 2005). However, in melanoma we did not detect interaction of Brn3a with p53 in co-immunoprecipitation experiments. Instead of release of p53 from interaction with Brn3a, p53 accumulation and DNA damage was observed. Therefore, our data support a model in which accumulation of p53 protein is observed as a consequence of activated DNA damage signalling.

With respect to tumourigenesis, it is attractive to investigate Brn3a in the context of known oncogenic proliferation pathways. Elevated RAS/RAF signalling due to oncogenic mutations in BRAF or NRAS is well studied in melanoma, but these mutations are also common in non-proliferating senescent nevi (Pollock et al, 2003). In cell culture, expression of oncogenic BRAF in melanocytes induces senescence (Michaloglou et al, 2005). Brn3a, however, was very frequently expressed even in early stage melanoma cell lines, but absent in nevi and may be important to attenuate BRAF-induced senescence. Supporting this, a cooperative effect of Brn3a and RAS in malignant transformation has been shown in rat fibroblasts (Theil et al, 1993). DNA double-strand breaks due to DNA replication stress have been implicated in oncogene-induced senescence (Bartkova et al, 2006; Di Micco et al, 2006) and active RAS/RAF signalling can lead to tumourigenesis when p53 is inactivated (Dankort et al, 2007; Patton et al, 2005; Serrano et al, 1997; Yu et al, 2009). Indeed, Brn3a clearly reduced BRAF-induced DNA damage and senescence in melanocytes. However, Brn3a was not able to fully revert ^V600E^BRAF-driven senescence. Other known senescence pathways like DNA replication stress or metabolic stress are likely to be still active. Another reason may be the experimental setting where ^V600E^BRAF and Brn3a were expressed using the same promoters. This leads to similar protein amounts, but may lead to different protein activities, because Brn3a represents the wild-type form and BRAF the constitutively activated mutated form. Nevertheless, Brn3a was able to reduce BRAF-induced DNA damage in normal cells and DNA damage has also been observed in melanoma cells upon Brn3a inhibition. These cell lines harbour the ^V600E^BRAF allele and the DNA damage may reconstitute oncogene-driven DNA damage leading to a senescence-like phenotype. Together, Brn3a may play a key role in tumourigenesis by avoiding DNA damage caused by oncogene-induced stress. The potential roles of Brn3a in tumourigenesis are summarized in Fig 7G.

The identification of Brn3a in melanoma as a new tumour-promoting factor may have important impact for clinical applications. Brn3a may prove useful as a biomarker to distinguish early stage melanomas from benign lesions and its potential as a therapeutic target has been demonstrated in our study. Its tissue-restricted expression profile may allow therapeutics to target melanoma tissue more selectively. Chemical compounds, able to compete with Brn3 transcription factors, may provide the basis for Brn3a-specific inhibitors (Peixoto et al, 2008). An important issue that we pursue in the future is the characterization of the molecular mechanisms by which Brn3a contributes to malignancy. Brn3a and its associated signalling pathways may offer new approaches for the understanding of melanocytic transformation and for therapeutic interventions of this highly therapy-resistant tumour.

## MATERIALS AND METHODS

### Reagents and antibodies

Anti-caspase 3, anti-caspase 8 (1C12), anti-caspase 9, anti-Bcl-x_L_, anti-Bcl-w, anti-phospho-Chk2 (Thr68), anti-phospho p53 (Ser15), anti-phospho p53 (Ser20), anti-Bak and HRP-conjugated secondary antibodies were obtained from New England Biolabs (Frankfurt, Germany), anti-cytochrome *c* (clone 7H8.2C12) from BD Biosciences (Heidelberg, Germany), and anti-Brn3a (14A6), anti-Bax (3D2), and anti-p21 (F-5) from Santa Cruz Biotechnology (Heidelberg, Germany). Anti-Bcl-2 (Ab-1), anti-p53 (Ab-6) and anti-Mre11 were from Merck Biosciences (Schwalbach/Ts., Germany), anti-β-actin (AC-15) from Sigma (Taufkirchen, Germany) and anti-Rad50 (clone 2C6) antibody was from Millipore (Schwalbach, Germany). Primers and siRNAs were from MWG Biotech (Ebersberg, Germany).

### Cell culture

Human melanoma cell lines were a kind gift of Dr. M. Herlyn (Wistar Institute, Philadelphia, PA, USA) and maintained in a culture medium consisting of MCDB153 (Sigma) with 20% Leibovitz's L-15 (PAA Laboratories, Coelbe, Germany), 2% FBS (PAA Laboratories), 1.68 mM CaCl_2_ (Sigma) and 5 µg/ml insulin (Sigma). For analysis, cells were detached with 0.2% EDTA in PBS. Keratinocytes, fibroblasts and melanocytes were isolated from neonatal human foreskins and cultivated as described (Berking et al, 2001).

### Small interfering RNAs

siRNAs were designed as described (Besch et al, 2009). Sequences of the 19nt sense strand of each siRNA are: Brn3a 1: GCAAGAGCCAUCCUUUCAA; Brn3a 2: CCACGUACCACACGAUGAA; AND P53: GUACCACCAUCCACUACAA. Non-silencing control siRNAs were designed to contain random sequences that do not match within the human and murine genome. The sequences of control siRNAs were GCGCAUUCCAGCUUACGUA (named control or control 1) or GCGCUAUCCAGCUUACGUA (named control 2). siRNAs were transfected at 20 nM with 1.25 µl Lipofectamine RNAiMAX. In co-transfection experiments each siRNA (20 nM) was complexed separately to 1.25 µl Lipofectamine RNAiMAX.

### RNA extraction and quantification

Total RNA was extracted from cells using Trizol (Invitrogen) and analysed by quantitative RT-PCR. One microgram of RNA was reverse transcribed using Expand Reverse Transcriptase (Roche Diagnostics, Mannheim, Germany) and poly(dT) oligonucleotide (Roche Diagnostics). Quantitative PCR for Brn3a mRNA shown in Fig 1A was performed using the FastStart SYBR Green Master Kit (Roche Diagnostics). Specific amplification was controlled by melting curve analysis, gel electrophoresis, and additionally by sequencing of the PCR product. All other qPCRs were carried out using the LightCycler TaqMan Master Kit (Roche Diagnostics) together with the Universal ProbeLibrary system (Roche Diagnostics). Relative gene expression was expressed as a ratio of the expression level of the gene of interest to that of hypoxanthine phosphoribosyltransferase (HPRT) RNA determined in the same sample.

### Protein preparation and analysis

Adherent and supernatant cells were lysed in 50 mM Tris; pH 7.4, 0.25 M NaCl, 1 mM EDTA, 0.1% Triton X-100, 0.1 mM EGTA, 5 mM Na_3_VO_4_, 50 mM NaF and protease inhibitors (Complete, Mini, EDTA-free, Roche Diagnostics). Lysates were analysed by enzyme-linked immunosorbent assay (ELISA) for ATM activity (R&D Systems, Wiesbaden-Nordenstadt, Germany) or by immunoblotting as described (Besch et al, 2009). Protein levels of β-actin were analysed as a control for constant loading and transfer. In Brn3a immunoblots using anti-Brn3a (14A6) antibody, PVDF membranes were denatured after protein transfer by incubating in a dilution series of 4, 2, 1, 0.5 and 0.25 M guanidine thiocyanate (Sigma) in 0.1% Tween 20 in PBS, followed by 10 min washing in 0.1% Tween 20 in PBS. Membranes were then blocked and further treated as described above.

### Gene reporter analysis

Brn3a transcriptional activity was tested using a firefly luciferase reporter plasmid (pGL2-Brn3a), kindly provided by Dr. E. Turner, University of California at San Diego, USA. It contains three consecutive Brn3a consensus binding sites linked to a prolactin minimal promoter (Gruber et al, 1997). Cells were transfected in 96-well dishes using FuGENE6 (Promega) with 50 ng of the reporter plasmid and 50 ng of a Renilla-Luc-expressing plasmid (phRLTK; Promega). Luciferase activity was measured according to the manufacturer's protocol (Promega). Luciferase activity values were normalized to Renilla activity of the same extract.

### Immunocytochemistry

Cells were fixed in room temperature with 4% paraformaldehyde in PBS for 20 min, permeabilized with 0.1% Triton X-100 (Sigma) in PBS for 20 min, and blocked with 5% BSA in PBS for 2 h. For analysis of Mre11/Rad50 foci, cells were then incubated with anti-Rad50 (1:1000) and anti-Mre11 (1:100) antibodies overnight at 4°C. For Brn3a analysis cells were incubated with anti-Brn3a (Biomol; 1:1000). After washing with 1% BSA in PBS, cells were incubated with fluorescence-labelled secondary antibodies (Alexa Fluor 488 Goat anti-mouse SFX (Invitrogen); NorthernLights Anti-rabbit IgG-NL557 (R&D Systems); both 1:250) for 2 h at room temperature. Cells were covered with Gel/Mount (Biomeda, Foster City, USA) and analysed using a Zeiss Axioskop (Zeiss, Munich) with Axiovision software. Mre11/Rad50-foci displaying cells were quantified by counting approximately 4500 cells per condition.

### Immunohistochemistry

Histopathologically confirmed tissue samples of primary melanomas and melanocytic nevi were collected at the Department of Dermatology at the Ludwig-Maximilian University of Munich. Informed consent was obtained from all patients and the experiments conformed to the principles set out in the WMA Declaration of Helsinki. Paraffin-embedded sections were deparaffinized in xylene and rehydrated in a graded series of isopropanol. Antigen retrieval was achieved by microwave in citrate puffer, pH 6.0 (Chemicon ICH Select, Temecula, Canada). Blocking of unspecific binding was performed with FCS/TRIS 20%. The primary antibody was a monoclonal rabbit IgG anti-human Brn3a (clone EP1972Y, Biomol, Hamburg, Germany) at a dilution of 1:50; the negative control was a normal rabbit IgG. Secondary antibodies were mouse anti-rabbit antibody (M0737, DAKO, Glostrup, Denmark) and rabbit anti-mouse antibody (Z0259, DAKO) combined with alkaline phosphatase anti-alkaline phosphatase (APAAP) immunocomplex (D0651, DAKO), *i.e.* soluble immunocomplexes prepared from calf intestinal alkaline phosphatase and monoclonal mouse antibody to calf intestinal alkaline phosphatase. Fast red (Sigma-Aldrich, Steinheim, Germany) was used as substrate and Mayer's haematoxylin as counterstaining. The sections were mounted in Kaiser's glycerol gelatine. Nerve fibres and smooth muscle cells stained positive for Brn3a and, therefore, served as internal positive control for successful staining. Tissue sections were inspected by two investigators (CB, RB).

### Quantification of viable cells

Viable cells were quantified in 12-well dishes utilizing a fluorimetric assay (CellTiter-Blue Cell Viability Assay, Promega, Mannheim, Germany). Viable cells with intact metabolism are determined by their ability to reduce cell-permeable resazurin to fluorescent resorufin. Medium was replaced with 375 µl of culture medium and 75 µl of CellTiter-Blue reagent. After 1 h incubation at 37°C fluorescence was measured.

### Cell cycle analysis

Cells were fixed in 70% ice-cold ethanol. RNA was degraded by digestion with 100 µg/ml RNAse A (Sigma) at 37°C for 15 min. Cellular DNA was then stained with 50 µg/ml propidium iodide (Sigma) and analysed by flow cytometry and ModFit LT software (Verity Software, Topsham, USA).

### Quantification of apoptotic cells and cell death

Adherent and supernatant cells were analysed by staining with FITC-labelled Annexin V (Roche Diagnostics) and propidium iodide (Sigma). Annexin V staining was performed according to the manufacturer's instructions. Propidium iodide was added to a final concentration of 0.5 µg/ml. Cells were analysed by flow cytometry and CellQuest software (Becton Dickinson, Heidelberg, Germany).

### p53 activity

p53 activity was measured by its DNA binding capacity using an ELISA system (TransAm ELISA; Active Motif, Carlsbad, USA). Nuclear protein extracts were prepared using the Nuclear Extract Kit (Active Motif). Briefly, cells were incubated in a hypotonic buffer for 15 min on ice. Then, detergent was added and nuclei were collected by centrifugation (14,000 rpm, 4°C, 30 s). Proteins were isolated from nuclei as described in the manufacturer's protocol.

**The paper explained**

PROBLEM:

Melanoma belongs to the most deadly types of skin cancers and arises from melanocytes, the pigment cells of the skin. Recently developed therapies are typically based on targeting the MAP kinase pathway, which is activated in melanoma due to mutations in BRAF or NRAS. These therapies display unprecedented response rates, but they are characterized by frequent resistance and they are available only for a subset of patients. Therefore, alternative approaches are needed to improve therapy options for melanoma patients.

RESULTS:

A common observation is that molecules involved in embryogenesis can be expressed in cancer cells which may be a way to acquire malignant properties. During embryogenesis, pigment cells and neuronal cells both derive from the neural crest. In this study, we screened for neural crest molecules in melanoma and identify the neural crest transcription factor Brn3a being highly expressed in melanoma, but not in melanocytes and benign melanocytic tumours. Inhibition of Brn3a causes DNA damage that leads to cell cycle arrest and apoptosis in melanoma cell lines and reduces tumour growth in mice. Furthermore, the oncogenic property of Brn3a is analysed. Brn3a promotes anchorage-independent growth of melanocytes, increases tumour growth of tumourigenic melanocytes and cooperates with mutated BRAF to avoid oncogene-induced senescence.

IMPACT:

The study identifies a new factor in melanoma that is frequently expressed and important for melanoma survival. Due to this essential role, Brn3a and Brn3a-regulated genes represent potential new targets for therapy. The cooperation of Brn3a with oncogenic BRAF points toward a critical role in tumourigenesis. BRAF mutations are also common in benign tumours in which Brn3a is absent and, therefore, Brn3a may represent an important second oncogenic insult required for malignant melanocytic transformation.

### Tumour engraftment and RNA treatment *in vivo*

PEI F25-LMW/siRNA complexes were prepared essentially as described (Werth et al, 2006). Briefly, 54 nmol (720 µg) of siRNA were dissolved in 4835 µl 10 mM HEPES, 150 mM NaCl, pH 7.4, and incubated for 10 min. 590 µl PEI F25-LMW (6.1 mg/ml) were dissolved in 4835 µl of the same buffer, and after 10 min pipetted to the siRNA solution and incubated for 1 h. The mixture was then aliquotted and stored frozen in 150 µl aliquots. Prior to use, aliquots were thawed and incubated for 1 h at room temperature. One aliquot (150 µl containing 10 µg complexed siRNA) was used per injection. 1205Lu cells (3 × 10^6^) in 150 µl PBS were injected subcutaneously into both flanks of athymic nude mice (nu/nu). When solid tumours were established after 13 days, mice were randomized into treatment groups. Treatment was performed by intraperitoneal injection of 0.77 nmol (10 µg) of PEI F25-LMW-complexed Brn3a-specific siRNA (Brn3a 2) or PEI-complexed control siRNA. Tumour volumes were monitored at each treatment (see figure). Eighteen hours after the last treatment mice were sacrificed and tumours removed and immediately snap-frozen. Tumours were homogenized in 1 ml Trizol and RNA was prepared as described above. Animal studies were performed according to the national regulations and approved by the Animal Care and use committee of the Regierungspräsidium Giessen or the Regierung von Oberbayern. All efforts were made to minimize the number of animals and their suffering.

### Lentiviral transduction of cells

Brn3a was cloned into modified pLenti6 vectors (Invitrogen). Lentiviral particles were generated in 293FT cells with the Virapower packaging system (Invitrogen). Supernatant lentiviruses were concentrated using Amicon-Ultra 100 kDa filters (Millipore). Cells were infected by a 1.25-fold concentrated virus supernatant and selected with Blasticidin. In senescence assays, melanocytes were transduced with Brn3a-encoding and ^V600E^BRAF-encoding lentiviruses and selected with Blasticidin. ^V600E^BRAF transduction efficiency was controlled by fluorescence microscopy analysing co-expression of GFP which was consistently above 90% (data not shown).

### Quantification of anchorage-independent growth

Anchorage-independent growth was assessed by cultivation of the cells in soft agar utilizing the CytoSelect Transformation Assay (BioCat, Heidelberg). Cells (5 × 10^3^) were seeded into soft agar according to the manufacturer's protocol and cultivated for 7–11 days. Colonies (>10 cells) were determined microscopically and quantified by counting. Cell growth was determined by quantifying the cells fluorimetrically by staining cellular nucleic acids with CyQuant GR.

### Analysis of senescence-associated β-galactosidase activity

Cells were fixed with 2% formaldehyde, 0.2% glutaraldehyde in PBS for 5 min at room temperature, washed with PBS and incubated at 37 °C for fourteen hours with the following senescence-associated β-gal stain solution containing 1 mg/ml 5-bromo-4-chloro-3-indolyl-β-d-galactoside (X-gal): 40 mM citric acid/sodium phosphate (pH 6.0), 150 mM NaCl, 2 mM MgCl_2_, 5 mM potassium ferrocyanide. Cells were then washed with PBS and analysed under a light microscope.

### Statistical analysis

Unless indicated otherwise, Student's *t*-test was used to assess the significance of mean differences. Differences were considered significant at a *p*-value of 0.05 or less, two-tailed.

## Supplementary material

As a service to our authors and readers, this journal provides supporting information supplied by the authors. Such materials are peer reviewed and may be re-organized for online delivery, but are not copy-edited or typeset. Technical support issues arising from supporting information (other than missing files) should be addressed to the authors.
