# Supplementary material for: The neural crest transcription factor Brn3a is expressed in melanoma and required for cell cycle progression and survival
Source: EMBO Mol Med. 2013 May 13;5(6):919–34. doi: 10.1002/emmm.201201862 (PMC3779452; doi:10.1002/emmm.201201862)
Supplement: Supplementary file 1 [file emmm0005-0919-SD1.pdf]

EMBO Molecular Medicine

The neural crest transcription factor Brn3a is expressed in melanoma and required for cell cycle progression and survival

Tobias Hohenauer, Carola Berking, Andreas Schmidt, Sebastian Haferkamp, Daniela Senft, Claudia Kammerbauer, Sabine Fraschka, Saskia Anna Graf, Martin Irmler, Johannes Beckers, Michael Flaig, Achim Aigner, Sabrina Höbel, Franziska Hoffmann, Heiko Hermeking, Simon Rothenfusser, Stefan Endres, Thomas Ruzicka, Robert Besch

Corresponding author: Robert Besch, Ludwig-Maximilian University

Review timeline:

Submission date:	10 August 2012
Accepted:	04 April 2013

Editor: Anneke Funk / Céline Carret

Transaction Report:

No Peer Review Process File is available with this article, as the authors have chosen not to make the review process public in this case.
