# Supplementary material for: The neural crest transcription factor Brn3a is expressed in melanoma and required for cell cycle progression and survival
Source: EMBO Mol Med. 2013 May 13;5(6):919–34. doi: 10.1002/emmm.201201862 (PMC3779452; doi:10.1002/emmm.201201862)
Supplement: Supplementary file 2 [file emmm0005-0919-SD2.pdf]

Supporting Information

The neural crest transcription factor Brn3a is expressed in melanoma and required for cell cycle progression and survival

Tobias Hohenauer, Carola Berking, Andreas Schmidt, Sebastian Haferkamp, Daniela Senft, Claudia Kammerbauer, Sabine Fraschka, Saskia Anna Graf, Martin Irmler, Johannes Beckers, Michael Flaig, Achim Aigner, Sabrina Höbel, Franziska Hoffmann, Heiko Hermeking, Simon Rothenfusser, Stefan Endres, Thomas Ruzicka, Robert Besch

Supporting Information contents

Supporting Figure S1.

Transcriptional activity of Brn3a and control experiments for the phenotype upon Brn3a inhibition ……………………………………………………………….………..2

Supporting Figure S2.

Characterisation of apoptosis induced by Brn3a inhibition…………………………...…4

Supporting Figure S3.

Additional p53 analyses …………………………………………………………….…5

Supporting Figure S4.

Brn3a promotes anchorage-independent growth in immortalised human melanocytes……………………………………………………………………………6

Supporting Figure S5.

Brn3a promotes malignant transformation of primary fibroblasts in vitro and tumour growth in vivo in HRAS-transformed NIH3T3 fibroblasts................................7

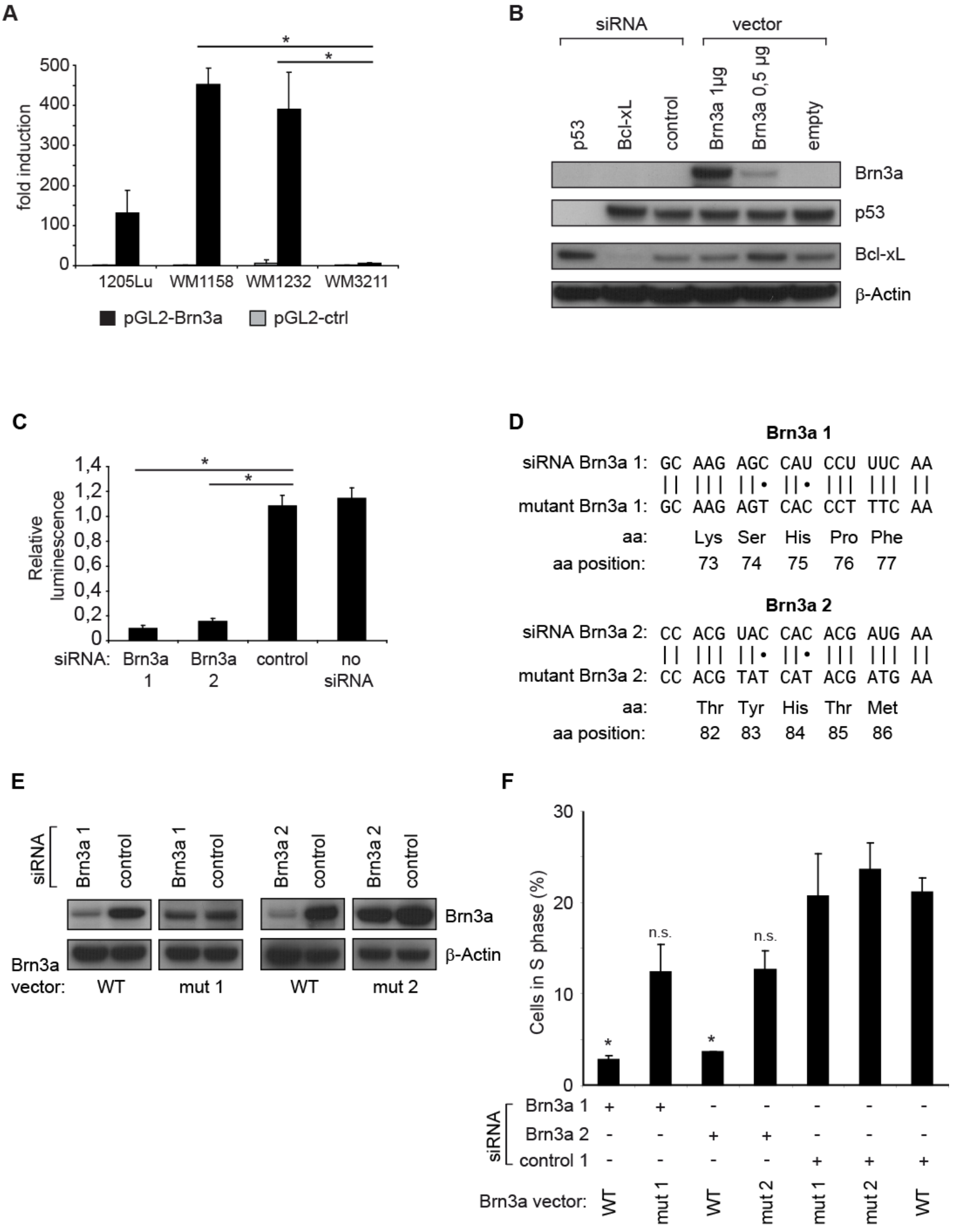
A
500
400
300
200
100
0
fold induction
1205Lu
WM1158
WM1232
WM3211
pGL2-Brn3a
pGL2-ctrl
B
siRNA
vector
p53
Bcl-xL
control
Brn3a 1µg
Brn3a 0,5 µg
empty
Brn3a
p53
Bcl-xL
β-Actin
C
1,4
1,2
1,0
0,8
0,6
0,4
0,2
0
Relative luminescence
siRNA:
Brn3a 1
Brn3a 2
control
no siRNA
D
Brn3a 1
siRNA Brn3a 1: GC AAG AGC CAU CCU UUC AA
mutant Brn3a 1: GC AAG AGT CAC CCT TTC AA
aa: Lys Ser His Pro Phe
aa position: 73 74 75 76 77
Brn3a 2
siRNA Brn3a 2: CC ACG UAC CAC ACG AUG AA
mutant Brn3a 2: CC ACG TAT CAT ACG ATG AA
aa: Thr Tyr His Thr Met
aa position: 82 83 84 85 86
E
siRNA
Brn3a 1
control
Brn3a 1
control
Brn3a 2
control
Brn3a 2
control
Brn3a
β-Actin
Brn3a vector:
WT
mut 1
WT
mut 2
F
30
20
10
0
Cells in S phase (%)
n.s.
n.s.
siRNA
Brn3a 1
Brn3a 2
control 1
Brn3a vector:
WT
mut 1
WT
mut 2
mut 1
mut 2
WT

Supporting Figure S1. Transcriptional activity of Brn3a and control experiments for the phenotype upon Brn3a inhibition.

A. Melanoma cell lines were transfected with a Brn3a luciferase reporter plasmid (pGL2-Brn3a) or a control plasmid (pGL2-ctrl) for 48 h and luciferase activity was analysed. Mean ±SD is shown. *$p < 0.019$ or less compared to WM3211 cells, *t*-test, $n = 3$ per group. Increases versus control vector (pGL2-ctrl) were significant in all cell lines ($p < 0.035$ or less, *t*-test, $n = 3$ per group).

B. The cell line WM3211, characterised by low Brn3a levels, was transfected with the indicated siRNAs or with a Brn3a-encoding vector. Proteins were analysed 48 h after transfection. The data show that WM3211 cells can be efficiently transfected with DNA or siRNA.

C. 1205Lu melanoma cells were transfected with Brn3a-specific or control siRNAs. 24 h after transfection, cells were transfected with Brn3a luciferase reporter plasmid (pGL2-Brn3a) and analysed 24 h after plasmid transfection. Mean ±SD is shown. *$p = 0.001$ for both Brn3a siRNAs, *t*-test, $n = 3$ per group.

D. Generation of siRNA-insensitive Brn3a mutants by exchanging two nucleotides within the siRNA binding site. The mutations were chosen to encode the wild-type protein sequence (silent mutations). For each siRNA a corresponding mutant form was generated (upper and lower panel). Dots indicate the mutated bases leading to mismatches of the siRNA. Amino acid (aa) sequence and position is indicated.

E. 1205Lu cells were transfected with wild-type (WT) or mutated (mut 1 or mut 2) Brn3a vectors as indicated. 24 h after vector transfection, Brn3a siRNA 1, Brn3a siRNA 2, or control siRNA were transfected. Immunoblot analysis was carried out 48 h after siRNA transfection. Representative blots ($n = 3$) are shown.

F. Cell cycle analysis by staining with propidium iodide and FACS analysis of 1205Lu cells treated as described in (**E**). Mean ±SD is shown. *$p < 0.05$ compared to all control siRNA-treated cells, *t*-test, $n = 3$ per group (n.s.: not significant, $p > 0.05$).

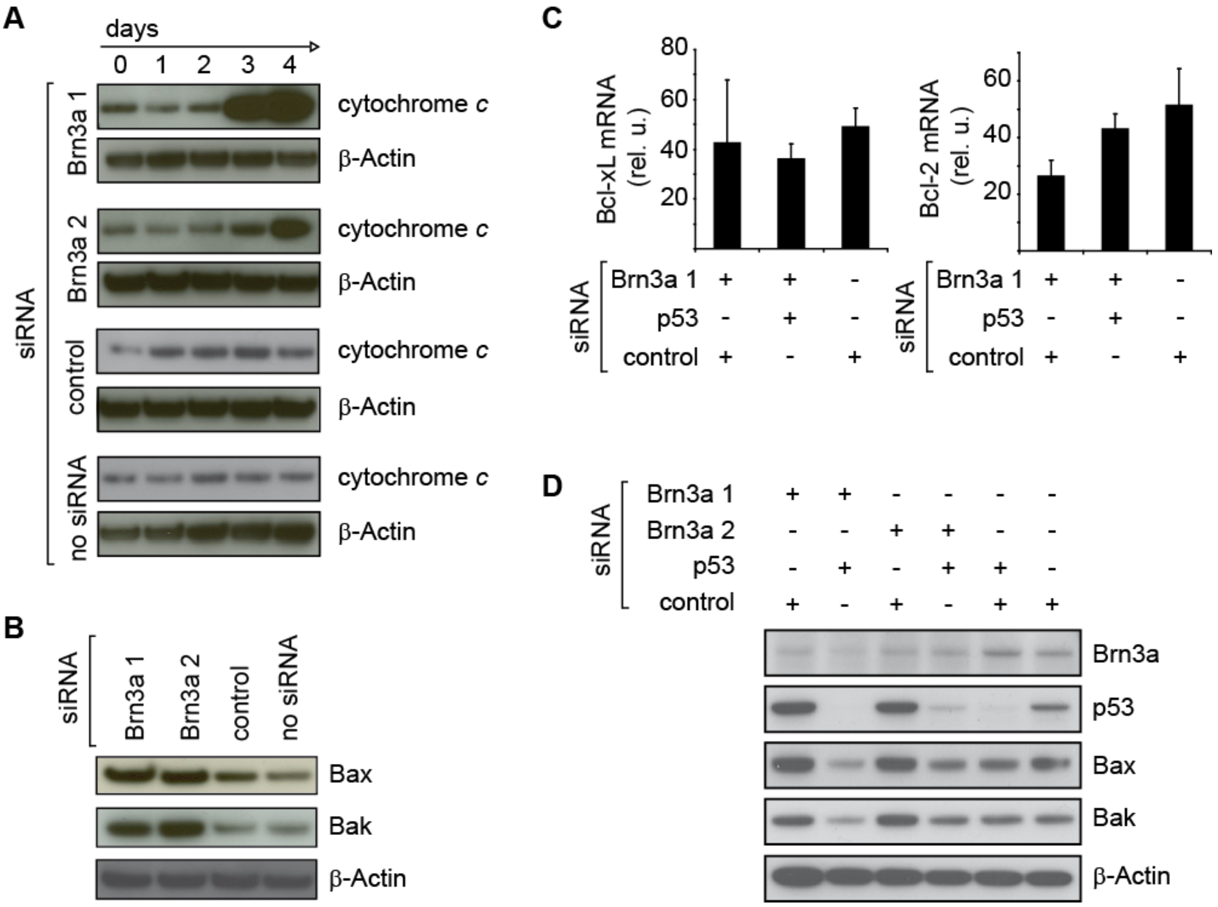

Supporting Figure S2. Characterisation of apoptosis induced by Brn3a inhibition.

A. 1205Lu melanoma cells treated with Brn3a-specific or control siRNAs were analysed for cytochrome *c* release. Cytosolic protein fractions were prepared at the indicated time points (1, 2, 3, and 4 days after transfection) using a digitonin-containing buffer (200 µg/ml in PBS) and analysed for the presence of cytochrome *c* in the cytosol by immunoblotting. Representative blots (n = 3) are shown.

B. Protein levels of proapoptotic Bax and Bak 3 days after transfection of Brn3a-specific or control siRNAs. Representative blots (n = 3) are shown.

C. Analysis of Bcl-2 and Bcl-xL mRNA levels in 1205Lu cells 48 h after co-transfection of Brn3a-specific, p53-specific or control siRNAs. Mean ±SD (n = 3) is shown.

D. Immunoblot analysis of the indicated proteins in 1205Lu cells 72 h after co-transfection of Brn3a-specific, p53-specific or control siRNAs (indicated by “+” and “-“). Representative blots (n = 3) are shown.

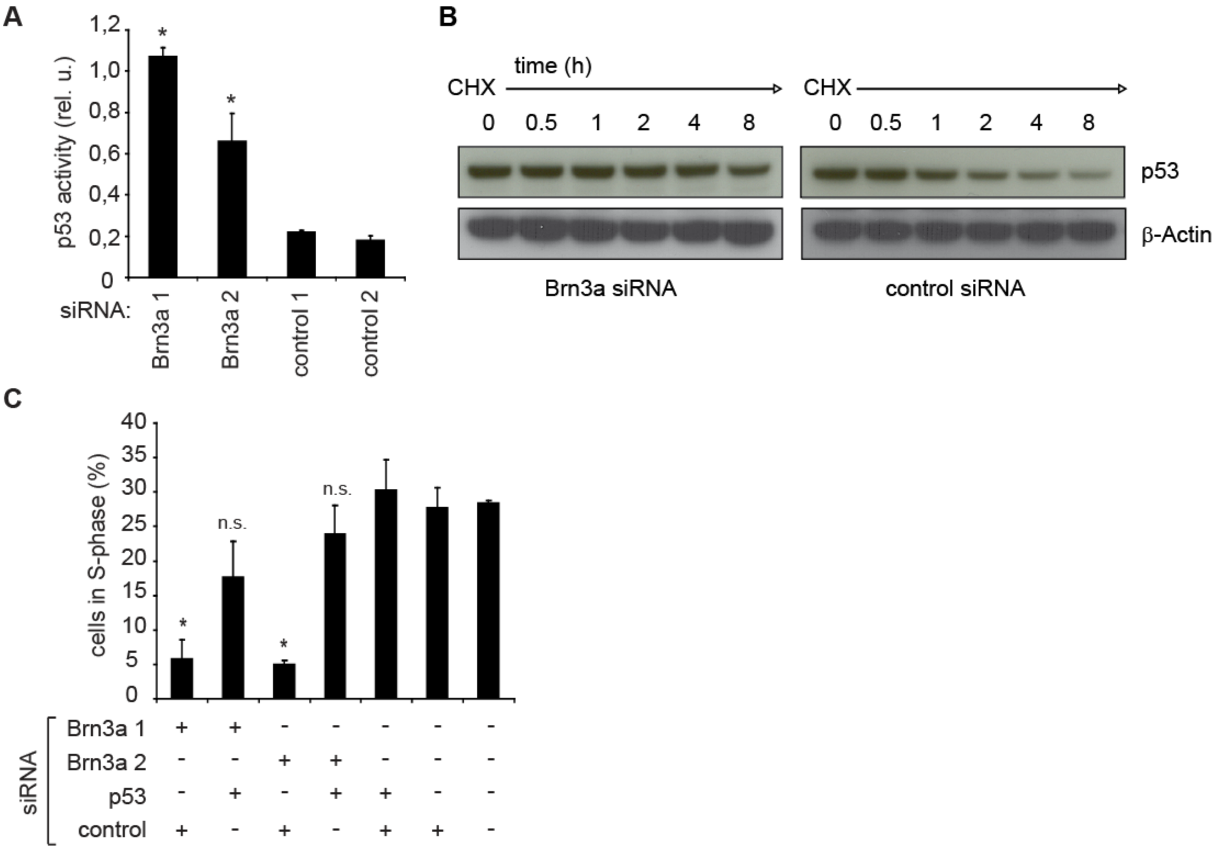

Supporting Figure S3. Additional p53 analyses.

A. Determination of p53 activity of 1205Lu melanoma cells 48 h after transfection of Brn3a-specific or control siRNAs. DNA binding capacity of p53 was quantified by ELISA. Mean ±SD is shown. *p = 0.03 or less compared to both control siRNA-treated samples, t-test, n = 3 per group.

B. 1205Lu melanoma cells were transfected with Brn3a siRNA 1 or control siRNA. 48 h after transfection, cycloheximide (CHX; 25 µg/ml) was added. Cells were analysed for p53 by immunoblotting at the indicated time points (0.5, 1, 2, 4, 8 h after cycloheximide addition). β-Actin served as a loading control. Representative blots (n = 3) are shown. Exposure times of Brn3a-siRNA and control siRNA treatments were adjusted to allow better comparison of p53 protein decay.

C. 1205Lu cells were transfected with Brn3a-specific, p53-specific or control siRNAs as indicated. Percentage of cells in the S phase was determined 48 h after siRNA treatment. Mean ±SD is shown. *p = 0.001 or less compared to control siRNA-treated cells, t-test, n = 3 per group; n.s.: not significant (p > 0.05).

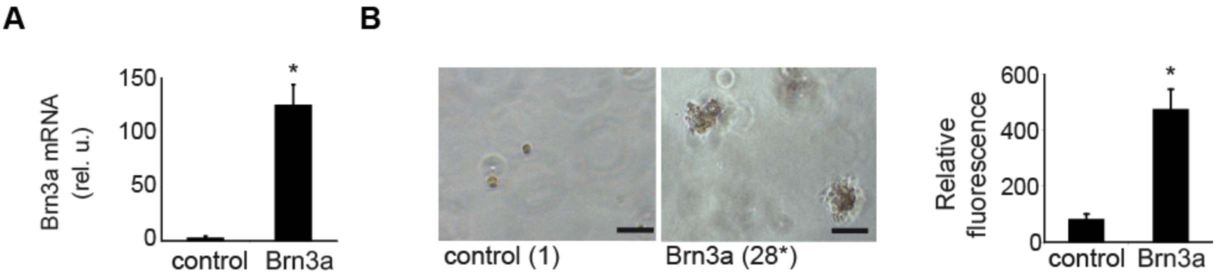

Supporting Figure S4. Brn3a promotes anchorage-independent growth in immortalised human melanocytes.

A. Expression of Brn3a in immortalised (p16 null, hTERT overexpressing) human melanocytes after lentiviral transduction of a Brn3a-encoding or an empty vector (control). Mean ±SD is shown. **p* = 0.001, *t*-test, *n* = 3 per group.

B. Left panel: Microscopic quantification of colony formation of immortalised melanocytes cultured for 9 days in soft agar. Numbers of colonies (>10 cells) are shown in brackets. **p* = 0.005, *t*-test, *n* = 3 per group. Right panel: Fluorimetric quantification of cellular DNA of cells cultured for 9 days in soft agar. Mean ±SD is shown. **p* = 0.008, *t*-test, *n* = 3 per group.

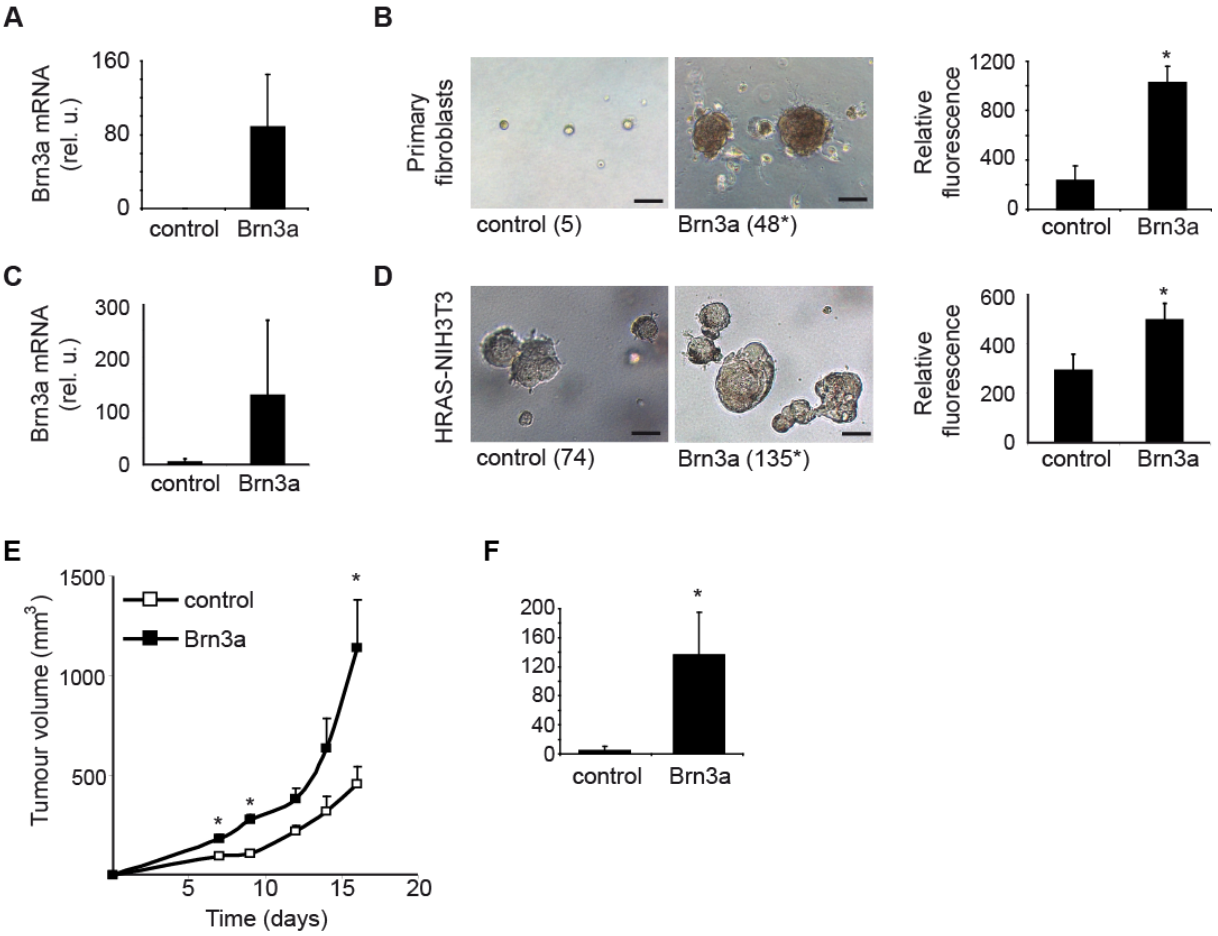
A
Brn3a mRNA (rel. u.)
160
80
0
control
Brn3a
B
Primary fibroblasts
control (5)
Brn3a (48*)
Relative fluorescence
1200
800
400
0
*
C
Brn3a mRNA (rel. u.)
300
200
100
D
HRAS-NIH3T3
control (74)
Brn3a (135*)
600
E
Tumour volume (mm^3)
1500
1000
500
Time (days)
5
10
15
20
F
200
160
120
40

Supporting Figure S5. Brn3a promotes malignant transformation of primary fibroblasts in vitro and tumour growth of HRAS-transformed NIH3T3 fibroblasts.

A. Levels of Brn3a in primary human fibroblasts after lentiviral transduction of a Brn3a-encoding or an empty vector (control). Mean ±SD (n = 2) is shown.

B. Left panel: Microscopic quantification of colony formation of primary fibroblasts cultured for 11 days in soft agar. Numbers of colonies (>10 cells) are shown in brackets. *p = 0.048, t-test, n = 3 per group. Right panel: Fluorimetric quantification of cellular DNA of cells cultured for 9 days in soft agar. Mean ±SD is shown. *p = 0.001, t-test, n = 3 per group.

C. Tumourigenic NIH3T3 fibroblasts were generated by stable transfection of the HRAS1 proto-oncogene and were further lentivirally transduced with Brn3a-encoding (Brn3a) or empty (control) vectors. Brn3a levels in HRAS-transformed NIH3T3 cells are shown . Mean ±SD (n = 2) is shown.

D. Left panel: Colony formation of HRAS-transformed NIH3T3 cells at day 7. *p = 0.025, t-test, n = 3 per group. Right panel: Fluorimetric quantification of anchorage-independent growth. Mean ±SD is shown. *p = 0.019, t-test, n = 3 per group.

E. Subcutaneous tumour growth of HRAS-transformed NIH3T3 fibroblasts transduced with Brn3a (Brn3a) or empty vector (control). Mean ±SEM is shown. *$p < 0.05$, t-test, n = 4 per group.

F. Brn3a mRNA levels in tumors at the end of the experiment. Mean ±SD is shown. *p = 0.02, t-test, n = 4 per group.
